# *Leishmania* Vesicle-Depleted Exoproteome: What, Why, and How?

**DOI:** 10.3390/microorganisms10122435

**Published:** 2022-12-08

**Authors:** Sofia Esteves, Inês Costa, Sara Luelmo, Nuno Santarém, Anabela Cordeiro-da-Silva

**Affiliations:** 1 Laboratory of Microbiology, Department of Biological Sciences, Faculty of Pharmacy, University of Porto, 4050-313 Porto, Portugal; 2 Institute for Research and Innovation in Health (i3S), University of Porto, 4200-135 Porto, Portugal

**Keywords:** *Leishmania*, leishmaniasis, exoproteome, extracellular vesicles, secretome, vesicle-depleted exoproteome, exosomes

## Abstract

Leishmaniasis, a vector-borne parasitic protozoan disease, is among the most important neglected tropical diseases. In the absence of vaccines, disease management is challenging. The available chemotherapy is suboptimal, and there are growing concerns about the emergence of drug resistance. Thus, a better understanding of parasite biology is essential to generate new strategies for disease control. In this context, in vitro parasite exoproteome characterization enabled the identification of proteins involved in parasite survival, pathogenesis, and other biologically relevant processes. After 2005, with the availability of genomic information, these studies became increasingly feasible and revealed the true complexity of the parasite exoproteome. After the discovery of *Leishmania* extracellular vesicles (EVs), most exoproteome studies shifted to the characterization of EVs. The non-EV portion of the exoproteome, named the vesicle-depleted exoproteome (VDE), has been mostly ignored even if it accounts for a significant portion of the total exoproteome proteins. Herein, we summarize the importance of total exoproteome studies followed by a special emphasis on the available information and the biological relevance of the VDE. Finally, we report on how VDE can be studied and disclose how it might contribute to providing biologically relevant targets for diagnosis, drug, and vaccine development.

## 1. Introduction

*Leishmania* spp. are protozoan digenetic parasites that have a complex life cycle. The parasite exists as motile flagellated promastigotes in susceptible sandfly vectors from the genus *Phlebotomus* (Old World) and *Lutzomyia* (New World). After a successful blood meal by a *Leishmania*-infected female sandfly, promastigotes arrive at the new host. Some will survive and differentiate into the amastigote form inside macrophages. The amastigotes persist and replicate in the phagolysosome of macrophages. The parasite proliferation and associated immune responses are responsible for a disease known as leishmaniasis, a vector-borne neglected tropical disease that threatens more than 1 billion people [1]. Without any human vaccine available, disease control relies on the effective diagnosis and treatment of diseased patients. The treatment options available present significant limitations related to activity, toxicity, and the emergence of resistant parasites. Therefore, new drug targets, vaccine candidates, and improved diagnostic approaches are needed to tackle the disease [2,3,4]. These problems are worsened by the zoonotic potential of the infection and the issues related to the implementation of One Health concepts [5]. Addressing these challenges requires improved knowledge of parasite biology and host interactions. Like any other pathogen, *Leishmania* has specific effector molecules that are essential to their survival that often contribute to disease. Due to the need to interact with the environment, these effector molecules will be either membrane-bound or released to the extracellular milieu. The protein content outside a cell in either conditioned medium or the extracellular matrix from environmental samples is generically known as the exoproteome [6]. The exoproteome includes actively secreted proteins and non-secreted proteins that can result from surface shedding or cell lysis. Thus, the ‘secretome’ and ‘exoproteome’ are not the same; the secretome is contained in the exoproteome, representing distinct overlapping concepts. The secretome is composed of proteins that are conventionally and unconventionally secreted [7]. Among the unconventionally secreted proteins, the proteins associated with extracellular vesicles (EVs) have been the subject of most studies in the last decade. In fact, since Judith Silverman’s report on the existence of EVs in *Leishmania donovani* [8], most studies involving exoproteome components in *Leishmania* spp. have focused on EV characterization; possible biological roles; and potentials for vaccine, diagnostic, and drug-target discovery. Considering the novelty of these studies, the several reviews that focused on this subject constitute a comprehensive overview of the available data on EVs’ fraction of the *Leishmania* exoproteome [9,10,11,12,13,14,15,16,17], and thus EVs will not be approached extensively in this review. The reason for this focus on EVs is anchored on the intrinsic properties of EVs [18]. The half-lives of these EVs can vary; sometimes they are broken down in a few minutes and circulate only in the extracellular space adjacent to the release, but they can also travel long distances and appear in many biological fluids such as urine or blood. This potential for persistency enables close-range and long-range signaling in a way that soluble agents cannot. Signaling-wise, they share many properties with soluble/surface agents and are also capable of delivering their content to target cells through fusion with the plasma membrane, phagocytosis, or receptor-mediated endocytosis. Although these characteristics of EVs are relevant, they are not the only agents for cellular interactions. For a long time, the total exoproteome was presented as the repository of these agents. Due to the novelty of the EV field, in *Leishmania*, the total exoproteome concept was mostly sidelined, with very few reports even using the term ‘Exoproteome’ or exploring the total exoproteome [19]. This was transversal to most microorganisms. More recently, some studies in bacteria began to compare the exoproteome with the outer membrane vesicles (bacterial EVs) containing a fraction [20,21]. This was the first step in revisiting the exoproteome potential. Still, these were mostly partially informative studies because the total exoproteome used to compare the EVs fraction still contained the EVs. In 2014, an approach was proposed to compare EVs with a non-EV fraction of the exoproteome in *Leishmania infantum* [22]. This concept was later exploited in other microorganisms such as *Pseudomonas aeruginosa*, *Candida albicans*, *Trypanosoma cruzi*, and *Acanthamoeba castellani* using a similar concept of a vesicle-free exoproteome [23,24,25,26,27,28]. This notion was also recently exploited, not in microorganisms but in amniotic mesenchymal stromal cells [29].

**Figure 1:**
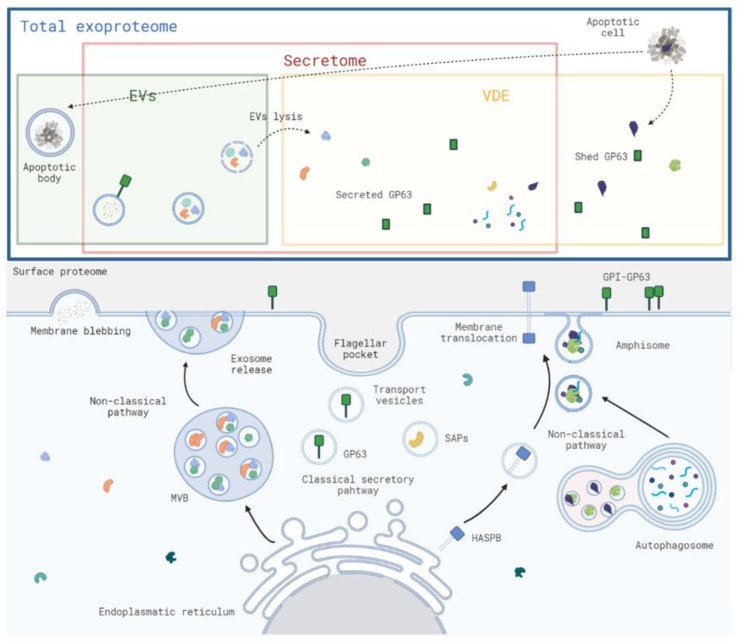
Schematic representation of the *Leishmania* spp. total exoproteome, secretome, extracellular vesicles (EVs), and vesicle depleted exoproteome (VDE) concepts. The exoproteome (blue rectangle) regroups all the secreted proteins as well as proteins that originated in the shedding of surface proteins or cell lysis; the secretome (pink rectangle) regroups all secreted proteins through conventional like the secreted acid phosphatases (SAPs) or non-classical pathways like the hydrophilic acylated surface protein B (HASPB); the EV fraction (green shaded rectangle) contains all vesicular components of the exoproteome, including those that originate from secretion, like the exosomes that are accumulated and released from the multivesicular bodies (MVBs) or from cellular lysis (dotted arrows); and the VDE (yellow shaded rectangle) contains all the non-vesicular components of the VDE, including those that originate from secretion, shedding from the surface, or lysis of cells or EVs (dotted arrows). The figure was created with BioRender.com.

The absence of comparative information between EVs and the non-EV fraction of the exoproteome is remarkable when considering that EVs are part of the exoproteome; the only comparative work available in *Leishmania* suggests that most exoproteome proteins in *Leishmania* under continuous growth conditions are not associated with the EVs [22]. The available data on bacteria and the related protozoa *T. cruzi* seem to support the same notion [21,23,26]. Even so, prediction data from the first study of EVs in *L. donovani* proposed a higher contribution of EVs to the total exoproteome (about half) [8]. Reasons related to the growth of the parasites in continuous or discontinuous approaches might contribute to these differences. Regardless, the most reliable, directly comparative evidence supports the fact that most proteins are present in the vesicle-depleted fraction (hereafter designated as VDE). This faction will be enriched in conventionally and non-vesicular unconventionally secreted proteins and other biologically relevant elements that can originate from surface protein shedding or the breakdown of other more complex structures (Figure 1). Their accumulation in the immediate vicinity of the parasite can contribute to specific biological functions that can include immune evasion and immune modulation. The scientific neglect associated with this essential part of the exoproteome prevents an in-depth comprehension of molecules that are intrinsically associated with parasite biology. The VDE is the logical option to find conventionally secreted and non-EV unconventionally secreted proteins. Their identification and clarification of their biological purpose are expected to be a major asset in the mitigation of the impact of leishmaniasis. Thus, in the present review, we will discuss the *Leishmania* exoproteome and describe how VDE studies in *Leishmania* spp. can be conducted as well as the associated challenges. We will also analyze the available VDE data for *Leishmania* and finally will finish with the latent potential of VDE studies.

## 2. Exoproteome

### 2.1. Definition

The term ‘exoproteome’ is used to describe the extracellular protein content in the vicinity of a biological system [6]. The exoproteome includes not only the conventional secretome but also all the most stable proteins present in the extracellular space, including proteins released from the surface or originating from cell lysis or EVs rupture (Figure 1). These proteins are a snapshot of how biological systems interact. The knowledge of the pathogen-derived exoproteome is thus essential for a better understanding of meaningful host–pathogen interactions. Several exoproteome studies in pathogenic and non-pathogenic organisms are available. The goals of these studies often went beyond the understanding of basic biology and had a strong focus on the search for and characterization of virulence factors (mostly through the comparison of strains with distinct intrinsic virulence). The most actively studied component of the exoproteome is the secretome; sometimes these terms are mistakenly used as synonymous. By definition, the secretome is the collective of the extracellular proteins released from an organism under defined conditions [30]. Therefore, secretome refers to the proteins actively secreted from the cell using a classical or a non-classical mechanism of secretion, including EV release. Thus, secretome is not synonymous with exoproteome. The exoproteome characterization in *Leishmania* is highly relevant because it might not only contribute to understanding how the parasite interacts with relevant cells but also enable the identification of relevant players in host–pathogen interactions [31].

### 2.2. Biological Sources of Exoproteome Components in Leishmania *spp*.

#### 2.2.1. Conventional Secretory Pathway

In *Leishmania*, most of the organelles involved in secretion and the early endocytic pathway are organized around the flagellar pocket. Similarly to higher eukaryotes, conventionally secreted proteins are associated with N-terminal signal peptides that direct the proteins through the endoplasmic reticulum (ER) [32]. In fact, despite having a very divergent eukaryotic lineage, the basic features of the classical secretory pathway are conserved in *Leishmania* [33] with the remarkable exception of the highly polarized delivery system focused around the flagellar pocket [34]. The flagellar pocket is a highly specialized structure present in all trypanosomatids. The known functions of this structure include the secretion of proteins, the addition of integral membrane proteins, and endocytic activity [35]. The membrane of the flagellar pocket represents 0.4–3% of the parasite surface [35] and is the most active endocytic organelle capable of internalizing an equivalent membrane area every 2 min [36,37]. In fact, this organelle was identified in trypanosomatids to be the endpoint of the secretion apparatus as was elegantly shown for the variant surface glycoprotein (VSG) in *T. brucei*. Using immunogold labeling, it was demonstrated that the VSG was sequentially present in the ER, Golgi cisternae, trans-Golgi network, transport vesicles, flagellar pocket, and ultimately the cell surface [38]. A few studies exist on the properties of individual conventionally secreted proteins. These proteins were shown to play specific roles in the survival and proliferation of the parasite in both the insect stage and in the mammalian host. In the insect vector, it is clear that several secreted elements have a significant role in the survival and proliferation of the parasite. The sandfly’s stomodeal valve is clogged with promastigote secretory gel (PSG) [39]. This PSG is secreted in such an abundance that it forms a semisolid plug in which the parasites are embedded, forcing the stomodeal valve open and enabling foregut colonization and transmission [40]. The PSG is mainly composed of filamentous secreted phosphoglycans [41]. These phosphoglycans were also shown to confer resistance to the fly midgut digestive enzymes [42]. These glycoproteins also seem to have a role in the establishment of an infection in the mammalian host [43]; due to their own immunomodulatory properties, they are attractive targets for vaccine approaches. In addition to these filamentous proteophosphogycans, this family of proteoglycans includes the promastigote-secreted acid phosphatases and other non-filamentous proteophosphoglycans from amastigotes and promastigotes. The promastigote-secreted acid phosphatases were among the first proteins studied in *Leishmania* [44]. In fact, for a long time acid phosphatase activity was considered a marker of virulence [45,46]. The acid phosphatases have species-specific characteristics. In *L. mexicana* they appear to be filamentous but *L. donovani* is not filamentous, although it is oligomeric [47]. Furthermore, this protein seems to be resistant to several proteases, which indicates that it can have a long functional life in the proteolytic environment of the sandfly midgut [48]. In addition, the amastigote non-filamentous proteophosphogycans have shown interesting properties in vitro by inducing vacuole formation in macrophages and also by activating the mannose-binding lectin pathway [49,50]. Another secreted protein important in the sandfly stage is the chitinase [51]. This protein is mostly studied in *L. mexicana* and was shown to be important in the survival inside the host. Its overexpression leads to faster sandfly colonization and damage to the stomodeal valve [52]. Interestingly the activity of this protein does not seem to be restricted to the sandfly stage. In fact, amastigotes present 2–4-fold higher levels of chitinase activity during their growth in vitro than promastigotes, which hints at a putative function in the amastigote stage [53]. Indeed, the overexpression of this protein leads to increased pathology in mice due to a higher intramacrophagic survival rate of the overexpressors [53]. GP63 also is expected to play a significant role in the sandfly vector because it is mostly produced in the promastigote stage [54] where it predictably should have a protective and nutritional role [55]. GP63 has been extensively studied in the interaction with the host cell and is involved in several mechanisms related to virulence [56]. Free or vesicle-bound GP63 can contribute to immediate protection against complement-mediated lysis [57] and might even enter the host cell’s cytoplasm through a lipid raft-dependent mechanism [58] to cleave the cytosolic host protein tyrosine phosphatases [58], thereby contributing to macrophage anergy [59].

#### 2.2.2. Unconventional Secretory Pathway

The classical secretory pathway is not the only way for the delivery of proteins into the extracellular space. Several proteins were reported to be exported without being involved with the ER–Golgi apparatus. The main features of unconventional secretion are: (1) a lack of known conventional signal peptides; (2) not requiring passage through the ER–Golgi apparatus; and (3) secretion resistance to brefeldin A, a specifical inhibitor of classical secretion [60]. The latter point has been used by some authors to support unconventional secretion in *L. donovani* [61], but several reports indicated that brefeldin A was not capable of disrupting conventional secretion in trypanosomatids [33,62]. Two main mechanisms have been proposed to explain the release of these proteins: the export mediated by direct translocation across plasma membranes or the transport through uncharacterized intracellular transport intermediates [63]. In *Leishmania* spp. a well-characterized unconventionally secreted protein called the hydrophilic acylated surface protein B (HASPB) has been described [64]. This protein is associated with the plasma membrane of metacyclic promastigotes and amastigotes [65] that are essential in the metacyclogenesis process [66]. Although the exact translocation process is still unknown, there are some important requirements for translocation. Using GFP SH4 chimeras, it was shown that the N-terminal SH4 domain phosphorylation regulates the subcellular localization of the protein [67]. In addition, the anchoring of HASPB to the membrane is dependent on specific myristoylation and palmitoylation of the same N-terminal SH4 domain [68]. There is now mounting evidence that these N-terminal SH4 domains indeed have a role in the translocation process. In 2007, it was shown that heterologous expression of these domains led to the reorganization of the membrane and induced non-apoptotic membrane blebs [69]. This latter fact was reminiscent of the membrane blebbing involved in the translocation of the non-classical secretion of interleukin 1 β [70]. An alternative delivery process for intracellular proteins consists of the fusion of amphisomes with the plasma membrane [71]. This process connects non-conventional secretion with autophagy. Macroautophagy and microautophagy, which are forms of autophagy that involve the trapping and degradation of complete regions of the cytosol by the lysosomal system [72], are thought to be major contributors to the amphisomes. Interestingly autophagy is a pivotal process in *Leishmania* development [73]. The macroautophagy of intracellular structures such as the glycosomes was described in *T. brucei* during its life cycle stage [74] and is also thought to occur in *Leishmania* [75]. Thus, this might be an active process in these parasites that contributes to the exoproteome. In 2010, a particular case of unconventional secretion was described for the first time in *L. donovani* with a release of vesicles similar to exosomes [8]. This component of the exoproteome has been extensively described elsewhere. The *Leishmania* EVs can have characteristics (density and size) that suggest the existence of multiple EVs subsets, similar to what occurs in mammalian cells [76]. This is a mostly unexploited facet of EVs. The content of exosome-like EVs might be different from the EVs that result from direct blebbing of the surface. The latter will have a surface that resembles the surface of the parasite, while the exosome-like EVs will probably have a distinct composition. The secretion of EVs can be induced by stress-inducing agents such as drugs or pH [8,22,77]. Although this field exploded in the last 10 years due to EV characterization, in different species of *Leishmania*, steps toward standardization are still required [77,78].

#### 2.2.3. Other Contributions to the Exoproteome Composition

Proteins released or shed from the surface of the promastigote are present in the exoproteome. These can be released by the action of proteases or phospholipases that release GPI-anchored proteins [56]. The surface of *Leishmania* spp. is composed of a few dominant components that are present in all species. Promastigotes are covered by glycoproteins that are anchored to the protozoan membrane by a GPI anchor. These proteins contribute to forming a glycocalyx that is about 7 nm thick in procyclic promastigotes; it thickens in later stages of development to at least twice as thick in metacyclic parasites. The dominant surface molecule of the promastigote is not a protein but is LPG. The most studied *Leishmania* protein is GP63. This metalloprotease is hidden under the 10-fold more abundant LGP that towers over all other surface components. GP63 expression is downregulated in amastigotes [79]. GP63 is one of the most prominent proteins in the parasite and is composed of two distinct forms: one that is conventionally secreted and another that is shed (released from the surface; probably by the processing of the GPI anchor). The dynamics of GP63 release are complex and known to be responsive to environmental stimuli [80]. The contribution from dead parasites or ruptured EVs can also be relevant depending on the approach selected for the generation of conditioned media.

## 3. The Vesicle-Depleted Exoproteome

The exoproteome of *Leishmania* spp. is composed of proteins that are released from the surface, secreted, and originated from cell lysis (Figure 1) [31]. For many years, the explanation for intracellular proteins without secretion signals in exoproteome preparations was cell lysis contamination in preparations [81,82]. Eventually, unconventional secretion and EV release provided the explanation for these proteins in exoproteome preparations [7]. Most exoproteome proteomic studies in *Leishmania* have been directed to the total exoproteome or, in the last decade, to the characterization of EVs (Table 1).

Few studies in microorganisms have evaluated the contribution of non-EV components in the exoproteome [22,23,24,25,26,27]. The importance of non-EV studies is quite obvious; the use of the classical secretion pathway to deliver effector molecules in microbial pathogens is almost a trademark in pathogenesis. Extracellular vesicle release seems to be a conserved mechanism in all eukaryotes and might have a latent physiological justification related to cellular homeostasis. Although EVs have significant reported biological properties, most of the proteins released in EVs are not expected to have a specific function in the host or in the environment. On the other hand, the release of proteins through the classical secretory pathway is a major investment by an organism and is normally environmentally regulated. If there is a biological investment to produce and release a protein to the extracellular environment using conventional on non-EV unconventional pathways, we must infer the existence of a strong biological imperative. Therefore, non-EV secreted proteins should have a specific extracellular role; for pathogenic organisms, there might be evolutionary pressure toward virulence. Thus, focusing exoproteome studies only on EVs will neglect a significant source of biologically relevant elements. Interestingly, *Leishmania* research was at the forefront of these studies with a proteomic characterization in 2013 [22] and two subsequent functional studies [76,92]. To not consider the VDE ignores the fact that an organism invests significant resources in conventional secretion and also the contribution of surface-protein shedding.

### 3.1. Available Proteomic Information on Leishmania *spp*. VDE

The VDE can be considered the exoproteome depleted of EVs or the non-EV exoproteome. In the last years, EVs have been the focal point of most exoproteome studies in *Leishmania*. The available information on the VDE is scarce but clearly supports the notion that not only the EVs have relevant biological properties [76,92]. Several proteins identified in the *L. infantum* VDE are part of the most abundant proteins associated with the core proteome of seven *Leishmania* spp. (Table 2).

The VDE from stationary and logarithmic *L. infantum* is distinct from EVs recovered from the same parasites [22]. The differences are both qualitative and quantitative. Only 9% of the protein identifications were shared between the VDE and EVs recovered from the logarithmic; this number increased to 19% when stationary parasites were evaluated. The VDE of *L. infantum* is dominated by GP63 and fructofuranosidade. The VDE from logarithmic and stationary *L. infantum* promastigotes presented a GO profile that was distinct from the respective EV fraction. In addition, the amount of overlap with a lysed parasite proteome was greater in the EV fraction in comparison to the VDE. This highlighted the uniqueness of the VDE. The most abundant protein in the VDE was GP63. In stationary parasites it accounted for almost 25% of all detected spectra. The second most abundant protein was beta-fructofuranoside. This protein is probably involved in the hydrolysis of sucrose to glucose and fructose in the sandfly. Both of these proteins were also co-purified with the EVs. Considering their high abundance in the VDE we cannot exclude that their presence, through indirect association to the surface of EVs, might be similar to what was reported for corona proteins in EV preparations [93]. The VDE in *Leishmania* is enriched over time in peptidases and proteophosglycans [22]. The relevance of both these families cannot be understated, and they are mostly accumulated in VDE and not in EVs. Although the *Leishmania* VDE should contain the conventionally secreted proteins and also the free proteins that are secreted by EVs’ independent manner, the use of a secretion-predicting tool did not show a large increase in the identifications of these proteins.

### 3.2. VDE Recovery Approaches

#### 3.2.1. Cultivation Method

In recent years, great progress in mass spectrometry (MS), bioinformatics, and analytical techniques have enabled the broad-scale analysis of proteins in complex biological samples such as whole tissue or culture media [94]. The quick development of these combined techniques led to the birth of a significant interest in the exoproteome of *Leishmania* (Table 1) [31]. Although the proteomic techniques that have been developed are highly efficient, the detection of exoproteome components in culture supernatants presents specific challenges. The standard culture medium for the growth of *Leishmania* spp. is a complex mixture dominated by serum supplement components. The presence of serum proteins represents a specific problem in the detection of the parasite exoproteome because it will mask the parasite-derived components. In addition, proteins released into the medium upon parasite death might contribute very significantly to the exoproteome by masking the secretome. The low abundance of secreted proteins poses another challenge because concentration steps are required before a subsequent proteomic analysis. Two major techniques to resolve the concentration problem include selective precipitation or direct concentration using ultrafiltration. To overcome the above-mentioned limitations for exoproteome recovery, the most commonly used procedure consists of washing the parasites to eliminate the serum components and then incubating them in a serum-free medium for a defined time. There is a short interval between the time of culture required for obtaining detectable amounts of secreted proteins and the cell survival in a serum-free medium to avoid cell lysis, therefore short culture times are preferred. Studies of *Leishmania* spp. tended to use less than 6 h of incubation in serum-free media [62,77,89]. Some EVs studies conjugated a temperature increase to generate more EVs [91]. The evaluation of the VDE presents some added limitations that are not present in standard exoproteome studies. The growth of the parasites must be done in a protein-free medium for long-term cultivation or a minimal FBS-free medium for short-term culture. The use of an FBS-depleted medium, a common approach to recovering EVs, is not an option for the VDE. Protein-free medium options are available and should not present a limitation for the promastigote stage [95]. VDE studies in amastigotes are technically limited presently due to the intracellular requirements for bona fide amastigotes. When focusing on the promastigote form, we can highlight two types of cultivation methods to generate the conditioned medium containing the exoproteome: either the recovery of a conditioned medium upon continuous cultivation for a defined time frame, hereafter designated as a continuous approach; or a discontinuous approach that encompasses short periods of cultivation in a minimal medium to prevent parasite death. The only “in vivo” secretome study was the recovery of *Leishmania* EVs from the sandfly midgut; this constituted a continuous method of recovery because the EVs were recovered directly from the sandfly midgut lavages [88]. This watermark study not only demonstrated that EVs were released in vivo, but it also validated the generic EV composition found on in vitro studies with promastigotes. Presently, only one study has reported the effect of parasite cultivation in exoproteome component recovery using the same recovery method [22]. This study compared the EV and VDE fractions of the exoproteome using the continuous and discontinuous approaches. The main highlight of this study was the fact that the main impact of the cultivation was in the quantitative composition of the exoproteome fractions. Similar proteins were identified, but the relative amount of peptide spectra quantified was quite distinct. For example, the top three protein identifications in the continuous approach were GP63 (30% of total spectra), beta-fructofuranosidade (6% of total spectra), and beta-tubulin (5% of total spectra); for the discontinuous approach, they were alpha tubulin (14% of total spectra), histidine secretory acid phosphatase (9% of total spectra), and beta-tubulin (6% of total spectra). In the discontinuous approach, GP63 represented 0.9% of the total spectra detected. Thus, although both approaches could claim that GP63 was present, for one approach the GP63 was detected in much higher amounts. The same was true for beta-fructofuranosidade: this protein, which in continuous conditions is easily detected in MS in logarithmic and stationary parasites, represented 6% of the total identified spectra in both approaches but became almost completely lost in the discontinuous approach (0.3% of total spectra detected). This protein was characterized in 2015 as a highly conserved secretory invertase that is differentially expressed by promastigote developmental forms [96]. Interestingly, sucrase activity in *L. donovani* in extracellular media was reported in 1994, thereby lending support to the accumulation verified in the continuous approach [97]. The root of these differences between the continuous and discontinuous approaches might be found in the parasite digenetic life cycle. The parasite must be able to respond swiftly to dramatic environmental changes to quickly adapt to a new host. Most of the information available on this phenomenon came from EVs studies. There was an increase in EV secretion upon drug-induced stress, temperature shifts, or changes in pH [8,22,77]. This might be associated with the need to respond to environmental changes. Thus, the impact of the exoproteome recovery approach must be assessed because the composition and perceived biological effects might be distinct depending on the growth conditions and the time of culture. This fact is well known, and the standard method for promastigote culture maintenance is simple dilution. It is also important to state that in continuous conditions there might be an impact of the medium used. This is well established for standard cultivation [95]. Thus, if there is an impact on the exoproteome, and VDE, it remains to be evaluated. As expected from what was exposed above, there is also a clear impact on EVs’ proteomic composition depending on the options taken to produce the conditioned medium. The exoproteome recovered in discontinuous conditions is enriched in heat-shock proteins and stress-associated proteins such as trypanothione reductase. This is a sound biological option when considering that the digenetic lifestyle of the parasite requires extreme biological changes. The swiftest way to accomplish this would be the vesicle-mediated release of intracellular content. This would enable not only a faster remodel of intracellular metabolism but also pave the way to losing membrane for the differentiation from the larger promastigote into the smaller amastigote. For this, EVs similar to microvesicles would be the best option due to their expected release process when compared to that of EVs similar to exosomes. The EVs with different sizes and densities can be recovered in *Leishmania,* thereby suggesting distinct origins [76]. Considering these notions, the use of a continuous or discontinuous approach can be adequate for different goals. For the study of parasite biology associated with growth in vitro, the continuous approach produces relevant information that has been shown for logarithmic and stationary promastigotes of *L. infantum*. Using a discontinuous approach to characterize log and stationary parasites might not produce the most significant data because the main impact would be the medium change and the adaptation to new environmental conditions. The use of a discontinuous approach can also be adequate depending on the goals of the study and will also provide insights into *Leishmania* biology while considering the digenetic life cycle. Nevertheless, no approach will fit all goals.

#### 3.2.2. Vesicle-Depleted Exoproteome Recovery

**Figure 2:**
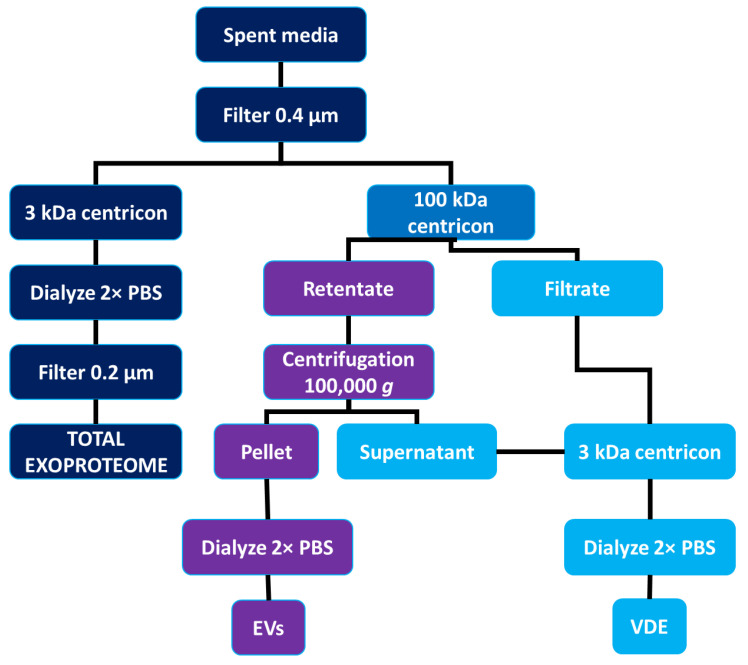
Recovery approach used to compare different exoproteome fractions as proposed in [76]. PBS, phosphate-buffered saline.

Considering the exoproteome’s complex nature, investigating the VDE-specific purification process steps are required (Figure 2).

The process requires the removal of EVs from the conditioned medium. To ensure this, a combination of ultrafiltration and ultracentrifugation was proposed. Both of these techniques can be used for the depletion of EVs from FBS [98]. The proposed approach is efficient in depleting EVs from the VDE, although it might lead to the loss of some VDE components that might be co-purified with the EV fraction. The process starts with the recovery of the conditioned medium and filtration to remove any remaining parasites. Then, by using ultrafiltration devices with a 100 kDa cut-off, the conditioned medium is separated into the retentate and filtrate containing low-molecular-weight proteins. The EVs containing retentate are then subjected to an overnight ultracentrifugation step that is similar to what is done for exosome removal from FBS. Without disturbing the pellet, the supernatant containing high-molecular-weight proteins is added to the previously recovered medium and is subjected to ultrafiltration using a 3 kDa cut-off. After concentration, this will constitute the VDE. Some free proteins might still be associated with the EV fraction, so further purification steps can be taken to ensure EV purity [77]. When studying the VDE it is essential to ensure that contaminating EVs are not present, thus we recommend using DLS or NTA in conjugation with TEM to confirm purity. The characterization of purity using Western blotting is possible using VDE or EV-specific proteins. Considering that only one study exists, the proposal of candidates for this purity assessment would be precocious. Still, the profile is clear: proteins that are only present in EVs and proteins only present in the VDE. A concern related to the VDE recovery process is the degradation of the protein content. This is more serious when considering that proteases are enriched in the VDE. To minimize this, low temperatures or even protease inhibitors can be used. Considering the nature of the approach, the molecular-weight cut-off of the filters can be adjusted to study the VDE enriched on proteins with specific sizes. It is important to state that this is not the only possible approach. Figure 2 depicts a possible strategy that can be optimized depending on the experimental setup and intended research goals.

### 3.3. VDE Research Potential

The VDE, much like other proteomic fractions recovered from the parasite such as the total proteome, the total exoproteome, or EVs, are valuable tools that can be used to understand parasite biology and advance the comprehension of the infectious process and disease. The discovery and characterization of the VDE might identify proteins relevant to diagnosis, vaccine development, and the understanding of host–parasite interactions that can contribute to the management of leishmaniasis.

#### 3.3.1. Diagnostics

The diagnosis of *Leishmania* infection and disease is essential for the implementation of adequate infection control measures. The available approaches for detecting infection are based on the visualization of parasites in infected tissue samples, the detection of genetic material, or through several serological approaches. Intracellular proteomes have been exploited with success to deliver proteins used in serological assays. Like any other proteomic sample, the exoproteome components are a logical source of antigens to exploit for serological and molecular use. In fact, this was proposed for the total exoproteome of *L. infantum* collected from logarithmic promastigotes at 26 °C for 6 h [86]. The serum from pooled symptomatic dogs presented a distinct reactivity pattern compared to that of the total parasite extract used as the control. This highlighted the immunogenicity and diagnostic potential of the total exoproteome. Considering that the promastigote VDE is enriched in antigens that are associated with promastigotes and even promastigote-specific secreted proteins, there is the potential to use it to screen for antigens that are found only in early stages of infection and that might be used for markers of exposition or early subclinical infections. For this purpose, the VDE is probably superior to other sources of readily assessable antigens such as the total extract of the parasites, total exoproteomes, or EV-enriched proteomes. The reasons for this possible superiority are related to the intrinsic nature of the exoproteome. Although the total exoproteome was exploited by some authors [86], the fact that it was a promastigote exoproteome might limit its use for diagnostics in comparison with other sources. Thus, for the discovery of antigens that might be interesting in detecting subclinical infections, the VDE holds the most promise. Transient responses to promastigote-specific secreted antigens will occur only during very early infections and might be used in conjunction with other serological responses to stratify animals in risk groups.

#### 3.3.2. Vaccine Development

The use of total exoproteome approaches in vaccine development has been proposed for several pathogens such as the bacteria *Staphylococcus aureus* or the parasite *Toxoplasma gondii* [99,100]. The lack of vaccines for human leishmaniasis is a serious limitation to mitigating the impact of the disease. One of the first vaccinal approaches was leishmanization [101]. A variation of leishmanization was the use of the exoproteome in vaccine approaches. This concept was exploited by considering that the *Leishmania* exoproteome is highly immunogenic and elicits strong immunity and some protection against infection in mice and dogs [102,103,104,105]. Eventually, these efforts lead to a vaccine for dogs: CaniLeish^®^ (Virbac, France). This vaccine is composed of purified excreted–secreted proteins of *L. infantum* produced in a serum-free culture system adjuvanted with QA-21, a highly purified fraction of the Quilaja saponaria saponin used for many years [106]. Although the efficacy was limited [107], it was still an example of the potential of the exoproteome. The high immunogenicity of the exoproteome makes it an attractive source of immunogenic antigens [108]. In fact, among the components of the possible recombinant vaccine Leish-111f, we found a protein isolated in the exoproteome: the thiol-specific antioxidant [109]. This vaccine has shown some potential in the context of human and canine leishmaniasis [109,110,111]. Intriguingly there was some species-specific cross-protection associated with the exoproteome: the *L. major* exoproteome was capable of protecting against *L. donovani* challenge but not against *L. braziliensis* [103]. The promastigote VDE of *L. infantum* was shown to exacerbate infection in BALB/c mice when administered together with promastigotes [76]. This may suggest that the inactivation of proteins present in the initial inoculum through vaccination-induced antibodies or potent cellular responses to these readily available antigens might contribute to decreasing the success of infection and limiting disease progression. In this context, the process of VDE generation will be crucial to enrich the VDE in proteins that are present in the initial inoculum. Notwithstanding this, several vaccine components were identified in the VDE. It is difficult to claim that the VDE would be a superior alternative to the total exoproteome or even EVs. Still, the VDE is a mostly untapped source of antigens that might be relevant in vaccine development.

#### 3.3.3. Understanding of Basic Parasite Biology and Host–Parasite Interactions

The total exoproteome studies that exist in the literature and those containing EVs support the study of the exoproteome as a means to understand parasite biology and exploit host–parasite interactions. *L. major*-secreted antigens were shown to be immunosuppressive by inducing IL-4 production in a lymphocyte culture [112], while the *L. mexicana* exoproteome induced inhibition of macrophagic functions via cleavage and activation of the host protein tyrosine phosphatases, specifically SHP-1 and PTP1-B [91]. The total exoproteome of *L. braziliensis* induces distinct activation profiles in macrophages. Strains isolated from self-healing cutaneous leishmaniasis presented a more inflammatory profile than a strain isolated from a disseminated form [83]. Although these data require confirmation in more isolates, this was a clear demonstration of the strain-specific effects that might be translatable into a better understanding of the disease. This fully justifies the search for species- and disease-specific exoproteomes in *Leishmania*. A significant advancement was made in a comparative total exoproteome study using seven distinct *Leishmania* spp. (*L. infantum*, *L. major*, *L. tropica*, *L. donovani*, *L. braziliensis*, *L. amazonensis*, and *L. tarentolae*) [84]. The data generated, which were produced in continuous culture conditions for 6 days, identified a core exoproteome of 306 proteins. Interestingly, this publication also reported 20 proteins that were conserved in the exoproteome of pathogenic *Leishmania.* In addition, species-specific proteins were identified, which opened the door to the study of disease-specific effectors. The differences reported must be confirmed in subsequent studies, but once again pointed to a diversity of the exoproteome composition that might contribute to specific disease presentations. The promastigote VDE already contains proteins that might have a relevant role in the survival inside the sandfly such as GP63, the proteophosphoglicans, or the beta-fructofuranoside. The promastigote VDE is also present in the early stages of infection; its study might contribute to a better understanding of infection establishment. The immunomodulatory properties of the *L. infantum* promastigote VDE, total exoproteome, and EVs were compared in two publications using an approach that compared the properties of the exoproteome fractions produced by an equivalent amount of parasites [76,92]. This option enabled the comparison of activity in more biologically relevant conditions in comparison with a simpler normalization by protein amount. Still, depending on the intended goal, other normalization options might be usable. In a comparative setting, the total exoproteome, VDE, and EVs presented a dose-dependent capacity to modulate cellular recruitment in air-pouch models and the capacity to diminish BALB/c bone-marrow-derived dendritic cells and macrophage responsiveness to secondary stimuli [76]. The immunological properties that were evaluated suggested that both the VDE and EVs were capable of inducing significant immunomodulation either through promoting or preventing cellular recruitment or through direct modulation of the target cell activation profile or responsiveness. Differences in the activity profile such as the reduced capacity of EVs do reduce the expression of surface markers CD40 compared to the total exoproteome and VDE, which highlights the need to study all exoproteome components. Even so, EV fractions and the VDE both contribute to the total exoproteome activity, thereby demonstrating that both EV-associated components and free proteins can contribute significantly to early events of infection and that they probably act with synergy. Similar observations of the total exoproteome, EVs, and VDE recovered from *L. infantum* promastigotes were presented in a publication related to the capacity to prevent blood-invariant natural killer T (iNKT) cell expansion and the disturbance of the capacity of these cells to respond to natural ligands [92]. Therefore, we can assume that many of the main immunomodulatory agents associated with *Leishmania* are present in the VDE and EVs. This is true for several significant immunomodulatory and infection-relevant proteins. The most described virulence factor in *Leishmania* is GP63. This protein has been the subject of comprehensive studies that have been reviewed extensively. In stationary *L. infantum* promastigotes, 76% of the spectra associated with GP63 were found in the VDE; the remaining 24% was detected in the EV-associated exoproteome fraction [22]. This demonstrated that GP63 can be present as free or EV-associated to exert the myriad potential biological roles attributed to it. The presence of free non-EV-associated GP63 or EV-associated GP63 can contribute to performing specific biological roles. Interaction with the complement and other early events such as the deactivation of intracellular transcription factors might be performed by the free non-EVs associated with GP63. In fact, access to the cytosol possibly is easier for free GP63 in the early stages of infection, but after sequestration of the parasite in the phagolysosome EVs associated with GP63 possibly can more easily escape the phagosome through increased fusion events associated with the increased acidity and membrane mobility of the phagosome. The elongation factor 1α (EF-1α) is another example of a VDE or EV-associated protein that is abundant in the exoproteome (Table 2) with known immunomodulatory properties. This protein’s expected biological role is associated with the binding of aminoacyl tRNAs to ribosomes, but EF-1α still was able to promote macrophage deactivation via activation of the Src homology 2 domain-containing tyrosine phosphatase-1 [113]. In stationary *L. infantum,* 47% of the spectra associated with EF-1α were found in the VDE; the remaining 53% was detected in the EV-associated exoproteome fraction [22]. Interestingly, in logarithmic promastigotes, this was more eschewed toward EVs: 88% of the spectra were associated with EVs. Thus, EF-1α seems to be mostly released through EVs, which would fit with the mostly intracellular role that is attributed to this protein. Recently, EVs have been proposed to be involved in drug-resistance mechanisms [87,114]. The properties of EVs for detoxification and their potential for involvement in the transmission of resistance genes make them appealing candidates. These properties are less applicable to the VDE. Moreover, the total exoproteome has been exploited in other microorganisms as a source of information to understand resistance. Changes in patterns might be associated with metabolic adaptations or the overexpression of proteins. This information might be relevant not only to understanding resistance but also to drug development. This notion was already exploited for other pathogens. Heavy metal exposure in *Candida sp.* isolates led to the release of stress-specific proteins [115]. In *S. aureus*, the comparison of exoproteomes also highlighted changes in the relative abundance of cytoplasmic proteins that might be a new feature of methicillin-resistant *S. aureus* [116]. In *Leishmania,* the first steps in this direction were given in an amphotericin-B-resistant isolate of *L. donovani* [117]. Thus, the VDE can also contain clues about resistant mechanisms especially if they are associated with the overexpression of proteins that might be detected in abnormal amounts in the VDE. This would be more relevant when considering that amphisome-driven unconventional secretion might be occurring in *Leishmania*. This would enable the detection of cytosolic and organelle-specific proteins in the VDE without the interference of EVs.

## 4. Conclusions and Future Perspectives

The non-availability of risk-free and efficacious chemotherapy options together with the lack of suitable vaccine approaches limits the control of leishmaniasis. Thus, a renewed knowledge of parasite biology and host–parasite interactions are warranted. The study of the exoproteome has been a reliable source of vaccine candidates and diagnostic markers for many pathogens. The study of the VDE as a source of conventionally secreted proteins and of non-EVs for non-conventionally secreted proteins is a promising pathway toward finding new agents. The information generated complements the EV data, thereby permitting a higher understanding of *Leishmania* biology. Ultimately, the advancement of studies containing all relevant players, the total exoproteome, EVs, and the VDE can play a significant role in the years to come. To completely explore the potential of the VDE and exoproteome, standardization steps similar to the efforts that are ongoing in the EV community [118] are required to enable a better comparison between different data sets.

## Figures and Tables

**Table 1:** Exoproteome studies involving proteomic characterization in *Leishmania* spp.

Species	Total Exoproteome	EVs	VDE
*Leishmania braziliensis*	[83,84,85]		
*Leishmania donovani*	[62,86,87,88]	[8]	
*Leishmania infantum*	[84,86]	[22,87,89]	[22]
*Leishmania major*	[84]	[88]	
*Leishmania tropica*	[84]		
*Leishmania amazonensis*	[84]	[90]	
*Leishmania tarentolae*	[84]		
*Leishmania mexicana*	[91]		

EVs: extracellular vesicles; VDE: vesicle-depleted exoproteome.

**Table 2:** Presence of VDE proteins identified in the most abundant core exoproteome proteins that were detected in promastigotes from 7 different *Leishmania* spp. (*L. infantum, L. major, L. tropica, L. donovani, L. braziliensis, L. amazonensis,* and *L. tarentolae*).

Most Abundant Core Exoproteome Proteins ^1^	Detected in VDE ^2^
14-3-3 protein-like protein	Yes
Adenosylhomocysteinase	
Cytoplasmic tryparedoxin peroxidase	Yes
Elongation factor 1-alpha	Yes
Elongation factor 2	Yes
Enolase	Yes
Heat-shock protein 83-1	Yes
Histone H4	
Leishmanolysin (GP63)	Yes
Nucleoside diphosphate kinase	Yes
Peptidyl-prolyl cis-trans isomerase	
Peroxidoxin 2	
Probable eukaryotic initiation factor 4A	Yes
Prostaglandin f2-alpha synthase (Fragment)	
Prostaglandin f2-alpha synthase/D-arabinose dehydrogenase	Yes
Putative 2,4-dihydroxyhept-2-ene-1,7-dioic acid aldolase	Yes
Putative beta-fructofuranosidase	Yes
Putative calpain-like cysteine peptidase	Yes
Putative heat-shock protein hsp70	Yes
Putative small myristoylated protein-1	
Putative small myristoylated protein-3	
Thiol specific antioxidant	
Tryparedoxin peroxidase	Yes
Tubulin alpha chain	Yes
Tubulin beta chain	Yes
Ubiquitin-60S ribosomal protein L40	

^1^ The proteomic information for this list was obtained from [84]. ^2^ The proteomic data for the VDE was obtained from [22].
